# Effects of Near-Freezing Temperature Combined with Jujube Polysaccharides Treatment on Proteomic Analysis of ‘Diaogan’ Apricot (*Prunus armeniaca* L.)

**DOI:** 10.3390/foods12244504

**Published:** 2023-12-16

**Authors:** Zhipeng Wang, Wei Wang, Wei Li, Rui Yang, Yanbo Li, Lusi Zhang, Mengying Zhang, Xuewen Li

**Affiliations:** College of Food Science and Pharmacy, Xinjiang Agricultural University, Urumqi 830052, China

**Keywords:** apricot fruit, proteomics, cell wall degrading enzyme, cold storage

## Abstract

This study involved the extraction of polysaccharides from jujube for application in apricot storage. Although near-freezing temperature (NFT) storage is commonly employed for preserving fresh fruit, its effectiveness is somewhat limited. Incorporating jujube polysaccharides was proposed to augment the preservative effect on apricots. Our findings demonstrated that the combined use of NFT and jujube polysaccharides can maintain fruit color, and effectively inhibit decay. Additionally, Tandem Mass Tag (TMT) quantitative proteomic technology was utilized to analyze protein variations in ‘Diaogan’ apricots during storage. This dual approach not only markedly lowered the activity of polyphenol cell wall-degrading enzymes (*p* < 0.05) but also revealed 1054 differentially expressed proteins (DEPs), which are related to sugar and energy metabolism, stress response and defense, lipid metabolism, and cell wall degradation. The changes in DEPs indicated that the combined use of NFT and jujube polysaccharides could accelerate the conversion of malic acid to oxaloacetic acid and regulate antioxidant ability, potentially extending the storage lifespan of apricot fruit.

## 1. Introduction

In recent years, growing concerns about pesticide use and residues on fruits have led to increasing attention to the development of safer fruit preservatives that are effective but do not harm our health. Although reducing the temperature for fruit storage can cut losses caused by metabolism, this approach is usually insufficient to fully protect harvested fruits [1,2]. The ‘Diaogan’ apricot (*Prunus armeniaca* L.), a typical climacteric fruit, exhibits a concentrated harvesting period and rapid respiratory climacteric post-harvest. Improper storage and preservation can result in swift softening and deterioration of the fruit within a brief timeframe [3,4]. Storing fresh produce at a near-freezing temperature (NFT) is a preservation technique that maintains low respiration rates and other metabolic processes. Prior research has highlighted the advantages of the initial storage of fresh apricots at NFT in enhancing their quality [5].

During prolonged cold storage, the fruit undergoes softening, which is associated with ripening and softening in apricots related to cell wall degradation by cell wall degradation enzymes [6]. Recently, plant polysaccharides have become a new focus in the field of postharvest fruit due to their ability to regulate the metabolism of fruit by controlling respiration rate, extending shelf life, and improving the preservation of rigidity [1]. The application of exogenous plant polysaccharides in postharvest treatments has notably enhanced the preservation of various crops, including potatoes [7], mangoes [8], and strawberries [9]. Jujube polysaccharides (natural plant polysaccharides) are of interest, but their impact on the storage and preservation of apricot fruits, as well as their potential mechanisms, remain unclear.

Proteomics, an increasingly important analytical technique, has been progressively applied in fruit and vegetable preservation to decipher the mechanisms of different preservation methods at the protein level. Considering the complexity of fruit ripening and aging, a comprehensive assessment of the molecular processes in fruits using proteomics is crucial [10]. Recent advancements in this field have led to significant discoveries, such as the role of vacuolar membranes in apple fruit senescence [11], metabolic shifts in kiwifruit revealed through iTRAQ-based quantitative proteomics [12], and the exploration of salicylic acid’s antagonistic effects on ethylene, auxin, and glucose during pear fruit senescence [13]. Nonetheless, proteomic studies focusing on the regulatory mechanisms of fruit quality maintenance and the impact of cold storage are limited [14,15].

This study investigated the relationship between changes in cell wall characteristics and differential protein expression levels during storage from the perspective of cell wall metabolism and proteomics, with the aim of exploring the potential mechanism of combined treatment (NFT + jujube polysaccharides) to delay the senescence of apricot fruit.

## 2. Materials and Methods

### 2.1. Fruit Materials and Treatment

Apricots were post harvested from local orchards in Yining, Xinjiang, China. The fruit had a freezing point of −2 °C. After removing the field heat for 2 h, the harvested fruits (day 0) were randomly divided into two groups.

The jujube polysaccharides were prepared as described previously [16]. Briefly, the dried jujube powder sample was treated twice with distilled water in the proportion of 1:40 (g/mL) at 90 °C for 4 h. The aqueous extracts were concentrated under vacuum and mixed with five volumes of anhydrous ethanol for 12 h at 4 °C. After centrifugation at 4000× *g* for 10 min, the precipitates were collected and washed successively with ether, anhydrous ethanol, and acetone, affording the crude polysaccharides.

The fruit freezing point was −2 °C and stored for 49 d for weekly sampling. (1) NFT group: apricots were stored at NFT (−1.5 to −1 °C) with 85–90% humidity for 7 weeks and were separately sampled at 0 d and 49 d; (2) NFT + jujube polysaccharide group: fruits were stored at temperatures ranging from −1.5 to −1 °C with 1.5 g/L jujube polysaccharide soak treatment for 10 min and were separately sampled at 0 d and 49 d. The samples collected from different storages were immediately frozen in liquid nitrogen and stored at −80 °C for further analysis.

### 2.2. Cell Wall Metabolism Enzyme-Related Enzymes

A total of 2 g of frozen apricot tissue was homogenized in an ice bath with 4 mL of pre-cooled 95% ethanol at 4 °C and 12,000 rpm for 10 min. Subsequently, the supernatant was discarded, and another 4 mL of pre-cooled 80% ethanol was added, followed by centrifugation under the same conditions. The tissue was then mixed with 8 mL of an 8.8% NaCl solution containing 2.5% polyvinyl polypyrrolidone (PVPP). After centrifugation at 12,000 rpm for 20 min at 4 °C, the supernatant obtained was further centrifuged [5]. Polygalacturonase (PG) activity was determined using the method described by Yang et al. [17], with results expressed in U/g. Cellulase (CX) activity was detected using the method outlined by Win et al. [18], and the results are presented in units of mg·h^−1^·g^−1^. The activities of β-galactosidase (β-GAL) and β-glucosidase (β-Gul) were determined according to Hagerman et al. [19], with the results expressed as μmol·h^−1^·g^−1^ and mg·h^−1^·g^−1^, respectively.

Frozen apricot tissue (2 g), mixed with 1 mL of phosphate-buffered solution (pH = 7.5), was centrifuged at 1200× *g*, and the supernatant was stored at 4 °C. Pectin methyl esterase (PME) activities were determined following the method described by Liu et al. [20], with the results expressed as U/g.

### 2.3. Proteins Extraction

Apricot fruits were pulverized into powder using liquid nitrogen, and 0.1 g of this sample was combined with 1 mL of phenol extraction solution (0.25 M Tris-HCl, pH 8.0, 24% *w*/*v* sucrose, 0.1 M NaCl, 0.04 M EDTA·2Na, 0.01 M DTT), supplemented with proteinase inhibitors. The mixture was homogenized by pipetting and incubated for 10 min. Subsequently, 1 mL of phenol-Tris-HCl (pH 7.8) was added, and the supernatant was separated by centrifugation at 6000× *g* for 15 min, isolating the phenol layer. The protein in the supernatant was precipitated by adding 5 volumes of 0.1 M ammonium acetate solution and incubating at 20 °C for 24 h, followed by centrifugation at 4 °C and 12,000× *g* for 10 min. The pellet was sequentially washed with methanol and acetone. Next, it was maintained at 20 °C for 12 h and centrifuged at 4 °C and 12,000× *g* for 10 min to collect the sediment.

The sediment was dried at room temperature and redissolved in 10 mL of SDS Cracking solution (50 mmol·L^−1^ Tris-HCl, pH 7.2, 2% *w*/*v* β-mercaptoethanol, and 1 mmol·L^−1^ phenylmethylsulfonyl fluoride) for 3 h. The mixture was centrifuged at 12,000× *g* for 20 min, and the supernatant was collected and stored at −80 °C [21].

### 2.4. Protein Enzymatic Hydrolysis and TMT Labeling

A total of 100 μg of the protein samples were incubated with 0.5 μL of 10 mM dithiothreitol at 56 °C for 20 min, followed by cooling to 25 °C. Iodoacetamide was added to achieve a final concentration of 10 mM, and the mixture was thoroughly combined and incubated in the dark for 30 min. The proteins were then digested using trypsin (Promega, Madison, WI, USA) at a protein-to-enzyme ratio of 50:1 at 37 °C for 12 h, and the resulting peptides were stored at 4 °C.

For the labeling of peptides, the TMT Labeling Reagent Kit (Thermo Scientific, Rockford, IL, USA) was employed. The samples were mixed with 100 mM TEAB. TMT reagent vials were sequentially arranged from smallest to largest. Seventy μL of the enzymatic hydrolysates was reacted with the contents of one TMT reagent vial. Each TMT reagent was first mixed with 65 μL of acetonitrile, and then 25 μL of the sample was added, and the mixture was incubated with 5% hydroxylamine for 15 min.

### 2.5. LC-MS/MS Analysis

LC-MS/MS analysis was conducted using an EASY-nLC1200 system (Thermo Fisher Scientific Rockford, IL, USA). One microgram of each sample was loaded onto a reversed-phase C18AQ column (Reprisal Ur 120, 1.9 μm, 100 μm × 15 cm, Thermo Fisher Scientific, Rockford, IL, USA) at a flow rate of 0.3 μL/min. Mobile phase A consisted of 0.1% formic acid (FA) and 98% water (2% acetonitrile), while mobile phase B consisted of 0.1% FA in 80% acetonitrile (with 20% water). The flow rate of the mobile phase was maintained at 300 nL/min with a gradient duration of 90 min. The gradient profile for elution was set as follows: 2–5% B for 2 min, 5–22% B over 68 min, 22–45% B for 16 min, 45–95% B for 2 min, and a final hold at 95% B for 2 min.

MS scans were performed on a Q Exactive HFX Orbitrap mass spectrometer (Thermo Fisher Scientific, Rockford, IL, USA) equipped with a nanoelectrospray Flex-Ion source (Thermo Fisher Scientific, Rockford, IL, USA). The instrument settings were as follows: a full scan range of 350−1600 *m*/*z*, a mass resolution of 120,000, and an automatic gain control (AGC) target of 3 × 10^6^. The top 20 most intense ions were selected for fragmentation by higher-energy collisional dissociation (HCD) with a normalized collision energy (NCE) of 32%. MS/MS spectra were acquired with a mass resolution of 4500, an AGC target of 1 × 10^5^, and a maximum injection time (MIT) of 96 ms.

### 2.6. Data and Bio Informatics Analysis

Data interpretation and quantification were conducted using Marquand (version 1.6.15.0) on a Linux system (Debian 9). The database utilized in this study was “uniprot-Oryza-sativa. Festa”. Protein functional annotation was carried out using the Blast2GO program. Protein groups from three sets of ‘Diaogan’ apricot samples were compared pairwise. To minimize false peptide identification, only proteins with peptide confidence intervals (≥20) exceeding 95% and containing at least one unique peptide were considered significant. Proteins with a *p*-value < 0.05 and a fold change > 1.2 were identified as significantly different. Those exhibiting significant differences across all three parallel samples were classified as differentially expressed proteins (DEPs). Gene Ontology (GO) enrichment analysis for protein identification was conducted using the website http://geneontology.org/ (accessed on 12 December 2022). Additionally, the Kyoto Encyclopedia of Genes and Genomes (KEGG) pathway enrichment analysis was performed to elucidate functions, accessible at https://www.kegg.jp/ (accessed on 12 December 2022). Hierarchical clustering for visualizing different protein expression levels was performed using the heatmap. Heatmap was plotted by https://www.bioinformatics.com.cn (last accessed on 10 November 2023), an online platform for data analysis and visualization.

### 2.7. Statistic Analysis

The Tandem Mass Tag (TMT) labeling technique was employed to analyze three biological and technical replicates for each treatment. Sample stability was assessed using IBM SPSS Statistics 20 (IBM Corp., Armonk, NY, USA). Kyoto Encyclopedia of Genes and Genomes (KEGG) pathways were retrieved from the KEGG database. Other charts pertinent to the proteomics analysis were generated using Origin 8.5 (OriginLab, Northampton, MA, USA).

## 3. Results

### 3.1. Cell-Wall-Modifying Enzyme Activities

**Figure 1:**
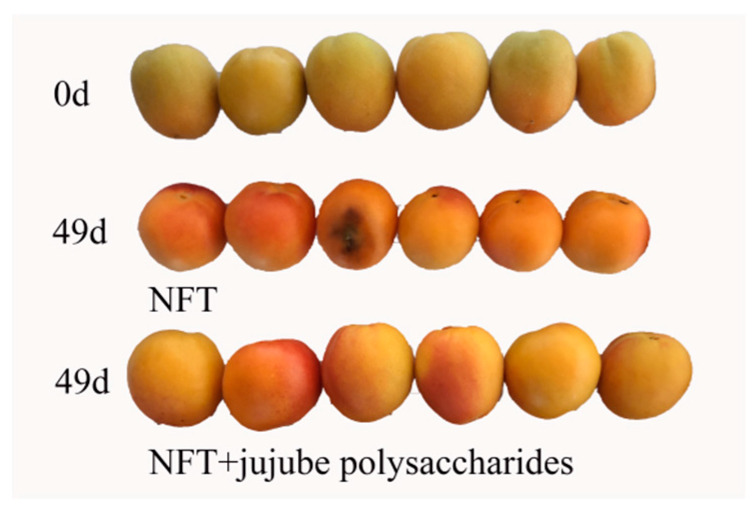
Appearance of apricot fruits stored in NFT and NFT + jujube polysaccharides at 0 d and 49 d.

**Figure 2:**
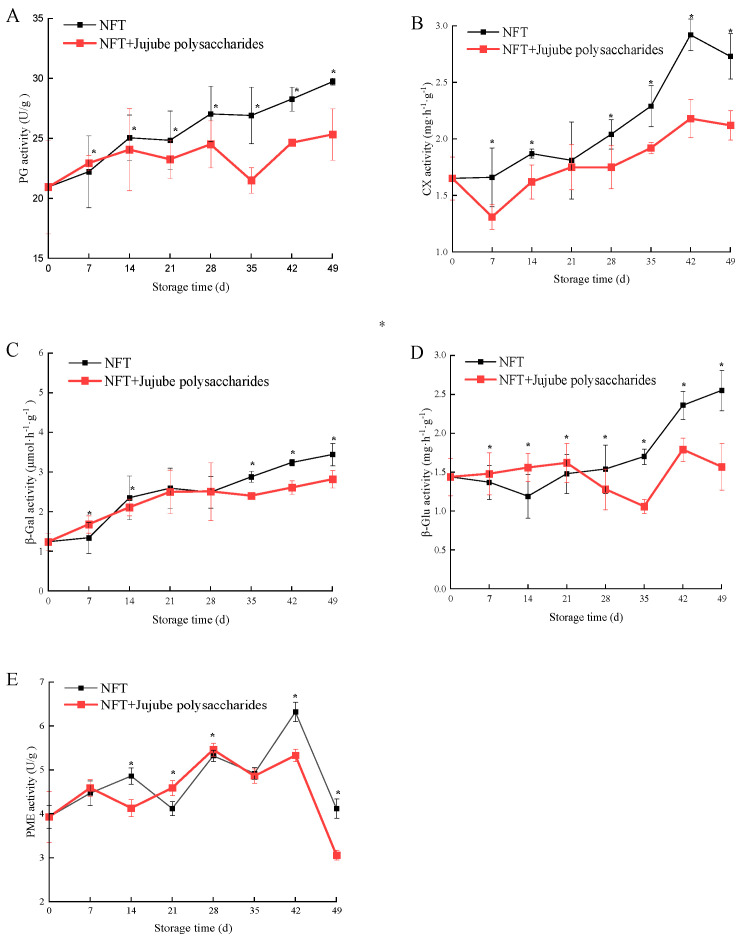
Changes in PG (**A**), CX (**B**), β-GAL (**C**), β-Glu (**D**), and PME (**E**) of NFT and NFT + jujube polysaccharides. Vertical bars represent the standard errors of the mean (*n* = 3). The symbol (*) within the storage time is significantly different (*p* < 0.05).

Figure 1 shows that the apricot samples stored in NFT and NFT + jujube polysaccharides can maintain fruit color and effectively inhibit decay. The implementation of NFT coupled with jujube polysaccharide storage emerges as a feasible strategy for extending favorable preservation outcomes. Figure 2A–E show an increase in the activities of PG, CX,β-Gal, and β-Glu throughout the storage period from 0 to 49 days. On day 49, the activities of PG, CX, β-Gal, and β-Glu in the NFT + jujube polysaccharide group were 14%, 22%, 18%, and 38% lower, respectively, than those in the NFT group (*p* < 0.05). Additionally, at the end of storage, the activities of PG, CX, β-Gal, and β-Glu were 1.1, 1.2, 2.27, and 1.09 times higher, respectively, in the NFT + jujube polysaccharide group compared to day 0. However, in the NFT group, these enzyme activities were 1.3, 1.6, 2.7, and 1.77 times higher, respectively, at the end of storage. The activity of PME fluctuated throughout the storage period in both groups. On day 49, the PME activity in the NFT + jujube polysaccharides group was 25% lower than that in the NFT group (*p* < 0.05).

### 3.2. Principal Component Analysis (PCA)

**Figure 3:**
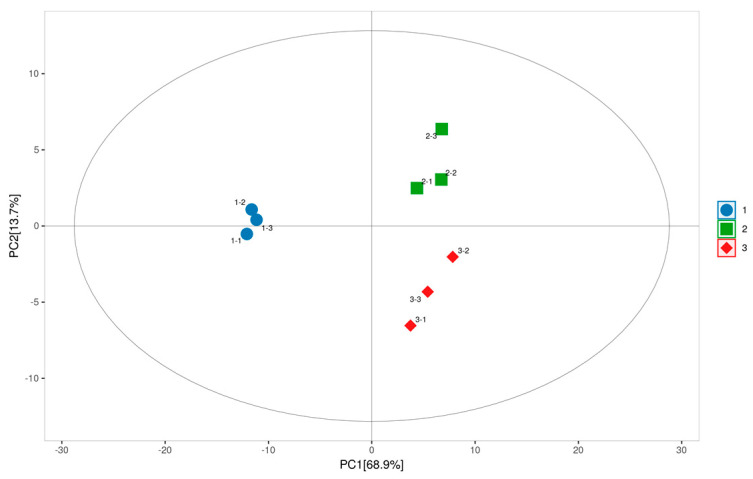
Score scatter plot for PCA model: “1” represents samples of storage at 0 d; “2” represents samples of NFT storage at 49 d; “3” represents samples of NFT + jujube polysaccharides storage at 49 d.

Prior to proteomic analysis, the semi-quantitative SDS-PAGE method was utilized to evaluate the protein samples. The results confirmed that a sufficient quantity of protein was extracted, and the purity of the samples was appropriate for subsequent experiments. Given that the SDS-PAGE method provides only semi-quantitative insights, a TMT-labeled proteomic approach was employed to identify differentially abundant proteins. A total of 7701 proteins were identified, of which 7550 credible proteins were selected for quantitative analysis after excluding data with any missing quantified values. The PCA revealed distinct separations between different storage stages of ‘Diaogan’ apricot samples based on the identified proteins. The PCA score plot (Figure 3) indicated that the first principal component (PC1) accounted for 68.9% of the total variance, while the second principal component (PC2) contributed 13.7%, collectively explaining 82.6% of the total variance within the 95% confidence interval of the samples, delineated by Hotelling’s T-squared ellipse. The groups stored under NFT and NFT combined with jujube polysaccharides were distinctly separated. Each group, represented by three biological replicates, clustered closely, demonstrating the excellent stability and reproducibility of the proteomic dataset.

### 3.3. Identification of Differentially Abundant Proteins

**Figure 4:**
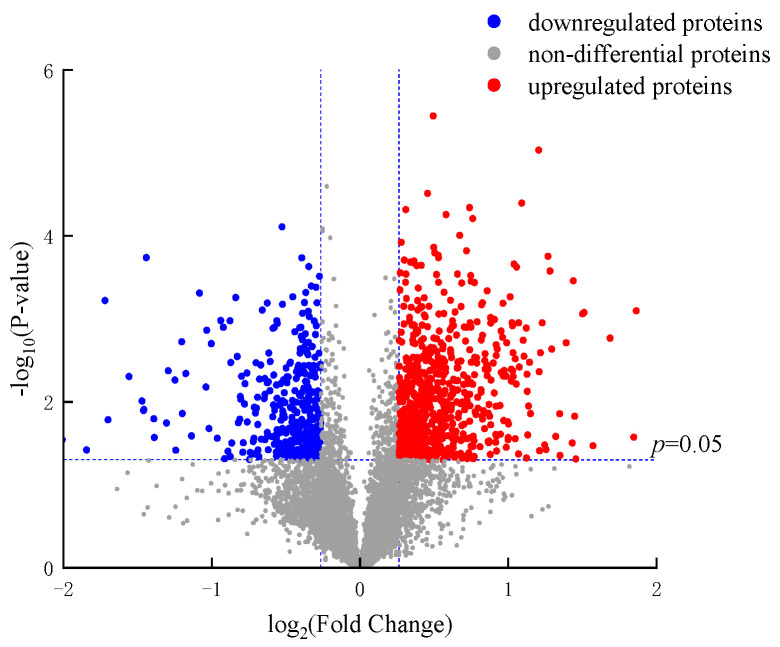
The volcano plot of 2 vs. 3. Proteins exhibiting significant upregulation in expression are represented in red, while proteins exhibiting significant downregulation in expression are represented in blue. Proteins with non-significant differences in expression are represented in gray.

**Figure 5:**
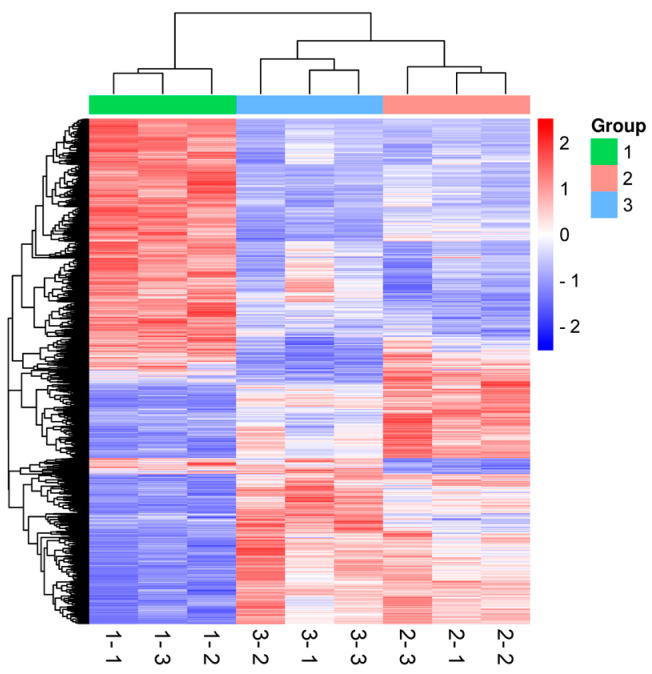
Hierarchical clustering analysis: “1” represents samples of storage at 0 d; “2” represents samples of NFT storage at 49 d; “3” represents samples of NFT + jujube polysaccharides storage at 49 d. The color key values ranged from 2 to −2. The x-axis and y-axis represent the multiples of the difference and significant difference (logarithmic transformation of 2 at the bottom), respectively.

The volcano plot provides a visual representation of the distribution of DEPs among the samples, and in Figure 4, it can be seen that there are 1054 DEPs between groups 2 and 3. Among these, 690 proteins were upregulated, and 364 were downregulation. Notably, as the number of DEPs increased, their significance also intensified. Hierarchical clustering was utilized to visually differentiate protein sub-groups and DEPs within these clusters, facilitating the quantification of proteins. Figure 5 indicates that proteins are color-coded to represent DEPs with distinct expression patterns within the protein sets. The color representation in group 1 is markedly different from groups 2 and 3. The significant color variations between groups 2 and 3 suggest that polysaccharide treatment in jujube influences protein expression during apricot storage differently, leading to varied storage effects. Some color discrepancies observed among the three replicates within each group might be attributed to individual variability among the apricots.

### 3.4. Bioinformatics Analysis

Gene Ontology (GO), a standardized international gene function classification system, was utilized to categorize the functional classifications of the DEPs in this study. All DEPs were annotated in accordance with the standard GO criteria. In the comparison between groups 2 and 3, the top 10 cellular components associated with the DEPs included parts of the chloroplast and plastid, chloroplast stroma, plastid stroma, ribosomal subunit, ribosome, plastid envelope, and small ribosomal subunit. The most prominent molecular functions comprised rRNA binding, structural constituent of ribosome, structural molecule activity, RNA binding, nucleic acid binding, electron transfer activity, phosphate ion binding, electron carrier activity, electron transfer within the cytochrome b6/f complex, and photosystem II activity, including large and small ribosomal subunit rRNA binding.

KEGG pathway analysis revealed that out of all identified proteins, a total of 4796 proteins were mapped to various KEGG pathways. In the comparison between groups 2 and 3, 100 DEPs were associated with 10 distinct pathways, displaying significant enrichment in 10 metabolic pathways (*p* < 0.05). The top five significantly enriched metabolic pathways for these DEPs were related to the ribosome, photosynthesis, pyruvate metabolism, fatty acid metabolism, and fatty acid biosynthesis.

## 4. Discussion

Over the years, the preservation of apricot fruit has remained a focal point of scholarly research. However, postharvest storage longevity of apricot fruit is limited. Extensive research has indicated that fruit softening is intricately linked to cell wall-hydrolyzing enzymes, which degrade cell wall polysaccharides and contribute to fruit softening through coordinated actions [22]. In this study, compared to NFT storage, NFT combined with jujube polysaccharides significantly inhibited the activity of PG, PME, β-Gal, CX, and β-Glu, thereby delaying the detrimental effects caused by the depolymerization of cell wall polysaccharides [23,24]. Storage temperature is a crucial factor influencing fruit ripening and senescence. Numerous studies have focused on the physiological and biochemical changes in apricot fruit under NFT storage. However, the use of jujube polysaccharides in fruit preservation has been relatively unexplored. Fruit ripening processes are complex and involve pathways unique to each fruit species. Therefore, in this research, we employed TMT proteomic technology to analyze protein expression during the ripening and senescence of apricot fruit under both NFT and NFT + jujube polysaccharide conditions.

Initially, our research focused on carbohydrate metabolism, a crucial process for energy production and conservation in apricot fruit. In this study, a majority of the identified DEPs were involved in carbohydrate metabolism pathways, including glycolysis, the tricarboxylic acid cycle (TCA), the pentose phosphate pathway, and Glyceraldehyde-3-phosphate dehydrogenase (GAPDH), a key enzyme in the glycolytic pathway [25]. Notably, a downregulation of GAPDH (Table S1) was observed in both the 2 vs. 3 and 2 vs. 1 group comparisons. This suggests that storage under NFT and NFT combined with jujube polysaccharides might similarly influence the apricot glycolytic pathway.

Cytosolic malate dehydrogenase (cy MDH) plays a pivotal role as a precursor in various metabolic pathways of fruit cells. It acts as a respiratory substrate in the mitochondrial tricarboxylic acid (TCA) cycle and can be converted to soluble sugars in the gluconeogenesis pathway [26]. Our findings revealed one upregulated protein (cy MDH) in the 2 vs. 1 group comparison. In contrast, an upregulation of cy MDH was noted in the 2 vs. 3 group, indicating that polysaccharides treatment potentially enhances the TCA cycle, accelerating the conversion of L-malic acid to oxaloacetic acid and facilitating NADH production [27]. Glucose-6-phosphate dehydrogenase (G6PD), involved in the initial step of the sub-pathway synthesizing D-ribulose 5-phosphate from D-glucose 6-phosphate (oxidative phase) [28], showed a general downregulation in our study, suggesting an inhibitory effect of NFT storage on the pentose phosphate pathway of glucose. These results support the hypothesis that NFT storage may preserve function through sustained metabolic activity [29].

Additionally, reactive oxygen species (ROS) are inevitable by-products of fruit ripening and senescence processes. The excessive accumulation of ROS, however, can lead to lipid peroxidation, membrane system damage, and oxidation of proteins and DNA, disrupting normal metabolism and ultimately causing cell death [30]. Plants have developed a comprehensive antioxidant system, including both antioxidant enzymes and metabolites, to mitigate oxidative damage. In this study, significant upregulations in the expressions of five peroxidases (POD), one catalase (CAT), two ascorbate peroxidases (APX), two superoxide dismutases (SOD), three glutathione peroxidases (GSH-PPx), and three glutathione transferases (GST) were observed in the 2 vs. 1 group. In the 2 vs. 3 group, there were noteworthy upregulations of nine POD, one APX, and one GSH-PPx. This upregulation of antioxidant enzymes suggests that jujube polysaccharides treatment can enhance the activity of these enzymes in apricots during cold storage, reducing ROS accumulation and maintaining redox homeostasis. Additionally, peroxidases can remove H_2_O_2_ and contribute to the cross-linking of cell wall components [31].

Heat shock proteins (HSPs) are crucial for enhancing plant stress tolerance and responding to stress. The accumulation of HSPs has been shown to protect tomato fruits from cold stress [32]. In this study, a small heat shock protein (HSP20) domain-containing protein was identified. In the 2 vs. 3 group, the expression of 6 HSP20 domain-containing proteins was upregulated. In the 2 vs. 1 group, seven proteins from the HSP20 family were upregulated, with one downregulated HSP20 protein. D’Ambrosio suggested that small HSPs were not deregulated, but rather heat shock cognate protein species (HSC70) were induced [33]. This suggests that jujube polysaccharides can induce the expression of HSP20, enhancing the stress resistance of apricot fruits during cold storage.

The softening of apricots post-harvest is primarily due to cell wall degradation, which leads to a reduction in pectin substances [34]. PG, PEM, β-Gal, and PEL are key enzymes involved in this degradation process [35]. Our study found that in the 2 vs. 3 group, there was a downregulation of one PEL, one PEM, one β-Gal, and one α-Gal. The downregulation of these enzyme activities indicates that NFT storage can delay fruit softening by inhibiting the expression of hydrolytic and glycoside hydrolysis-related proteins. Therefore, we infer that the treatment of apricots with jujube polysaccharides significantly inhibits the expression of PEM and β-Gal proteins, thereby delaying the degradation of pectin substances during cold storage [36].

Polygalacturonate-inhibitory proteins (PGIPs) are a type of defense protein associated with plant cell walls. They inhibit polygalacturonate enzymes (PGs) produced by pathogens and pests [37,38]. This study observed an upregulation of PGIPs in both the 2 vs. 1, and 2 vs. 3 groups. PGIPs, characterized by leucine-rich repeats (LRRs), specifically bind to cell walls and inhibit the activity of endogenous PGs. The interaction between PGIP and PGs facilitates the production and accumulation of oligogalacturonides (OGs) in plants, playing a vital role in plant defense signal transduction. This mechanism enhances plant disease resistance, effectively blocks pathogen invasion, and inhibits the onset and progression of related diseases [39].

Lipoxygenases (LOX) play a significant role in cellular processes, catalyzing the production of hydroperoxides from unsaturated fatty acids, and concurrently generating a substantial amount of reactive oxygen species (ROS). LOXs are involved in the peroxidation of cellular membrane lipids, leading to membrane damage and cell apoptosis [40]. At the end of storage, a downregulation of three LOX proteins was noted in the NFT storage group, whereas only one LOX protein was downregulated in the NFT + jujube polysaccharides storage group. This suggests that NFT combined with jujube polysaccharides can inhibit the upregulation of LOX activity during cold storage. Furthermore, the 2 vs. 3 comparison group exhibited a downregulation of one phospholipase C (PI-PLC) protein. Studies have linked increased PLC activity with membrane degradation induced by low-temperature stress [41].

## 5. Conclusions

During the ripening of ‘Diaogan’ apricots, there is a rapid softening and breakdown of cell wall components. The application of NFT combined with jujube polysaccharides effectively inhibits this cell wall disassembly. This inhibition is achieved by reducing the degradation of cell wall polysaccharides, which is evident in the decreased activities of enzymes such as PG, β-Glu, CX, β-GAL, and PME. Employing the TMT quantitative proteomic technique revealed 768 DEPs, showing more than 1.2-fold change in abundance, across 25 functional categories or biological processes. Key processes influenced during fruit storage and senescence include carbohydrate and energy metabolism, cell wall and membrane degradation, signal transduction, and stress response and defense mechanisms. Notably, NFT and jujube polysaccharide treatment effectively mitigates oxidative stress, likely due to the increased expression of antioxidant proteins such as POD, APX, and GSH-PPx. Identifying these functionally crucial proteins lays the groundwork for further exploration of the molecular mechanisms underlying apricot fruit storage, using genetic, transcriptional, and additional methodologies.

## Data Availability

Data is contained within the article or supplementary material.

## Supplementary Materials

The following supporting information can be downloaded at: https://www.mdpi.com/article/10.3390/foods12244504/s1, Table S1: List of some important differentially expressed proteins during apricot fruit storage at different manage.
