# Supplementary material for: Effects of Near-Freezing Temperature Combined with Jujube Polysaccharides Treatment on Proteomic Analysis of ‘Diaogan’ Apricot (Prunus armeniaca L.)
Source: Foods. 2023 Dec 16;12(24):4504. doi: 10.3390/foods12244504 (PMC10742872; doi:10.3390/foods12244504)
Supplement: Supplementary file 1 [file foods-12-04504-s001.zip › foods-2751349-supplementary.pdf]

Table S1. List of some important differentially expressed proteins during apricot fruit storage at different manage

2 vs 3 group

Accession	Protein description	Fold change	P
A0A6J5WID7	Peroxidase	1.30	0.036
A0A6J5V7Q2	Peroxidase	1.96	0.004
A0A6J5VBT9	Peroxidase	1.89	0.003
A0A6J5XTQ1	Peroxidase	1.46	0.002
A0A6J5UQS3	Peroxidase	1.6	0.003
A0A6J5XJ43	Peroxidase	2.1	0.004
A0A6J5X696	Peroxidase	1.89	0.002
A0A6J5TZ12	Peroxidase	1.27	0.000
A0A6J5UFQ9	Peroxidase	1.48	0.012
A0A6J5VXH3	Glutathione peroxidase	1.45	0.008
A0A6J5VJH9	L-ascorbate peroxidase	1.44	0.007
A0A6J5W818	SHSP domain-containing protein	2.07	0.033
A0A6J5V4L0	SHSP domain-containing protein	1.48	0.011
A0A6J5V0A3	SHSP domain-containing protein	1.43	0.041
A0A6J5WW61	SHSP domain-containing protein	2.73	0.015
A0A6J5XIV4	SHSP domain-containing protein	1.39	0.005
A0A6J5XVI5	SHSP domain-containing protein	1.77	0.002
A0A6J5XEL4	Pectate lyase	0.35	0.035
A0A6J5WSM1	Pectin esterase	0.25	0.003
A0A6J5W3J2	Alpha-galactosidase	0.39	0.028
A0A6J5WM85	β-galactosidase	0.23	0.004
A0A6J5UKD7	ADP-glucose pyro phosphorylase	0.71	0.035
A0A6J5Y9B6	ADP-glucose pyro phosphorylase	0.76	0.010
A0A6J5X150	Glyceraldehyde-3-phosphate dehydrogenase,	0.69	0.04
A0A6J5XB99	Glyceraldehyde-3-phosphate dehydrogenase,	0.69	0.006
A0A6J5X417	Malate dehydrogenase	1.53	0.002
A0A6J5TJC8	Glucose-6-phosphate 1-dehydrogenase	0.74	0.018
A0A6J5UKD7	ADP-glucose pyro phosphorylase	0.71	0.035

2 vs 1 group

Accession	Protein description	Fold change	P
A0A6J5XTQ1	Peroxidase	2.04	0.000
A0A6J5VAF6	Peroxidase	2.29	0.006
A0A6J5U4A7	Peroxidase	2.26	0.038
A0A6J5UQS3	Peroxidase	1.26	0.044
A0A6J5UQS3	Peroxidase	1.6	0.003
A0A6J5UV58	Glutathione peroxidase	2.43	0.001
A0A6J5W7N9	Glutathione peroxidase	1.87	0.001
A0A6J5Y859	Glutathione peroxidase	1.36	0.047
A0A6J5Y9J2	L-ascorbate peroxidase	1.26	0.031
A0A6J5XNS5	L-ascorbate peroxidase	1.47	0.035
A0A6J5TJT9	SHSP domain-containing protein	1.95	0.013
A0A6J5WMW3	SHSP domain-containing protein	3.33	0.019
A0A6J5WUP4	SHSP domain-containing protein	3.01	0.019
A0A6J5WSQ7	SHSP domain-containing protein	1.42	0.023
A0A6J5U3S4	SHSP domain-containing protein	3.05	0.041
A0A6J5V2B8	SHSP domain-containing protein	2.89	0.045
A0A6J5WWX1	SHSP domain-containing protein	1.45	0.011
A0A6J5X2N8	SHSP domain-containing protein	0.81	0.0444
A0A6J5XEL4	Pectate lyase	0.31	0.000
A0A6J5VX31	Pectate lyase	0.75	0.018
A0A6J5TYL3	Pectin esterase	2.27	0.000
A0A6J5W3J2	Alpha-galactosidase	0.80	0.014

A0A6J5UHJ7	Alpha-galactosidase	0.73	0.043
A0A6J5W3J2	Alpha-galactosidase	0.80	0.014
A0A6J5UHJ7	Alpha-galactosidase	0.73	0.043
A0A6J5VKQ7	β-galactosidase	1.61	0.028
A0A6J5UKD7	Glucose-1-phosphate adenylyl transferase	0.49	0.000
A0A6J5TNL3	Glucose-1-phosphate adenylyl transferase	0.54	0.001
A0A6J5X4V8	Glyceraldehyde-3-phosphate dehydrogenase	1.28	0.001
A0A6J5XKC4	Malate dehydrogenase	2.55	0.000
A0A6J5XHJ0	Malate dehydrogenase	1.71	0.001
A0A6J5TGG9	Malate dehydrogenase	1.32	0.007
A0A6J5X1B0	Glucose-6-phosphate 1-dehydrogenase	1.74	0.046
A0A6J5UKD7	Glucose-1-phosphate adenylyl transferase	0.49	0.000
A0A6J5TNL3	Glucose-1-phosphate adenylyl transferase	0.54	0.001
A0A6J5WNF4	Superoxide dismutase	2.04	0.003
A0A6J5X677	Catalase	1.47	0.001
